# Zoonotic arbovirus infections in cattle in Mozambique with special reference to Crimean-Congo hemorrhagic fever virus (CCHFV) and Rift Valley fever virus (RVFV)

**DOI:** 10.1186/s12985-025-02804-9

**Published:** 2025-06-06

**Authors:** José Fafetine, Teresa Cuinhane, Balal Sadeghi, Regina D. Miambo, Lucinda de Araújo, Martin H. Groschup, Ansgar Schulz

**Affiliations:** 1 Centro de Biotecnologia, Universidade Eduardo Mondlane (UEM), Maputo, Mozambique; 2 Institute of Novel and Emerging Infectious Diseases, Friedrich-Loeffler-Institut, Greifswald-Insel Riems, Germany; 3 Faculdade de Veterinária, Universidade Eduardo Mondlane (UEM), Maputo, Mozambique

**Keywords:** Mozambique, CCHFV, RVFV, Serology, Ticks, Arboviruses

## Abstract

**Background:**

Arboviruses pose a great threat to public health in sub-Saharan African countries. Mozambique is located in a region that is prone to climate change-related devastation, including heavy rainfalls and severe droughts that favor the emergence of zoonotic viruses transmitted by arthropods such as Crimean-Congo hemorrhagic fever virus (*Orthonairovirus haemorrhagiae*, CCHFV) and Rift Valley fever virus (*Phlebovirus riftense*, RVFV). Both viruses are closely associated with livestock farming, including cattle, and can cause symptoms of hemorrhagic fever in humans. Available previous data sets related to the presence of RVFV and especially CCHFV in Mozambique are rather scarce. Hence, the objective of this study was to evaluate the recent seroprevalence of both viruses in cattle in four localities of Limpopo National Park. In addition, ticks were collected and tested for the presence of different arboviruses.

**Methodology:**

A total of 460 cattle blood samples were collected and analyzed for the presence of CCHFV and RVFV antibodies using ID Screen CCHF Double Antigen Multi-species (IgM/IgG) and ID Screen Rift Valley Fever Competition Multi-species commercial ELISA test kits (IDvet, Grabels, France), respectively. 1176 ticks were collected from the same animals and analyzed with different RT-qPCRs assays for CCHFV, Nairobi sheep disease virus (*Orthonairovirus nairobiense*, NSDV) virus and Dugbe virus (*Orthonairovirus dugbeense*, DUGV). Selected ticks were further screened by using a pan-Flavivirus melting curve PCR.

**Results:**

The overall seroprevalence was higher for CCHFV (50%) compared to RVFV (28%). While a significant difference in seroprevalence between age groups was only found for CCHFV, there was a difference in RVFV seroprevalence between sampling sites that was not observed for CCHFV. None of the viruses tested were found inside the ticks.

**Conclusions:**

This study revealed the presence of anti-CCHFV and anti-RVFV antibodies in cattle from all four sampled localities suggesting that both viruses are circulating in cattle and may be an important cause of unidentified febrile illness in humans in the region.

**Supplementary Information:**

The online version contains supplementary material available at 10.1186/s12985-025-02804-9.

## Background

Arboviruses remain a significant public health threat in sub-Saharan Africa. Located in a region that is vulnerable to climate-related challenges, including heavy rains and severe droughts, Mozambique favors the emergence of zoonotic viruses transmitted by arthropods. Among those is Crimean-Congo hemorrhagic fever virus (*Orthonairovirus haemorrhagiae*, CCHFV), which is a widespread arbovirus that is endemic in Africa, southern Europe and large parts of Asia. Ticks of the genus *Hyalomma* represent the main virus reservoir and vector. Various domestic and wild animals can develop viremia after infection, although they do not show any clinical symptoms. Humans can be infected through the bite of infected ticks, contact with virus-containing blood/contagious tissue of viraemic host animals or CCHF patients, respectively. While many human infections are usually mild, there remains a risk of severe disease onset, including acute hemorrhagic symptoms and a high lethality rate [1]. On the African continent, South Africa in particular is considered a high-endemic area for CCHFV due to several annual human case reports and on the basis of a relatively large number of scientific studies within the past [2]. In sharp contrast, only limited confirmed information on the occurrence of the virus in the direct neighboring country of Mozambique exists. Apart from the first detection of CCHFV IgG antibodies in human sera in 2015, there have been neither serological findings in animals nor evidence of the virus in ticks in the country yet [2, 3].

Another important zoonotic agent in the country, which can lead to massive losses in livestock farming and infections in humans, is Rift Valley fever virus (*Phlebovirus riftense*, RVFV). RVFV is also an arbovirus, but in contrast it is transmitted via mosquito bites. Outbreaks have been described almost all over the African continent and the Arabian Peninsula. Local epidemics occur mainly after heavy rainfall and lead to severe economic losses due to abortions, milk yield decline and deaths in affected ruminants and camelids [4, 5]. Although, there is more information available on RVFV in Mozambique, the epidemiology of the virus is still obscure. The first report of the virus in the country was already in 1960 [6]. The last outbreak in livestock (sheep and goats) in Mozambique was in 2014 [7] and previous RVFV studies in cattle in Mozambique revealed overall seroprevalences of 37.3% in 2011 [8] and 36.9% in 2014 [9], respectively.

Apart from CCHFV and RVFV, there are other (less common studied) arboviruses that could occur in this region, such as the Dugbe virus (*Orthonairovirus dugbeense*, DUGV) and the Nairobi sheep virus (*Orthonairovirus nairobiense*, NSDV). DUGV and NSDV are both tick-borne viruses and genetically closely related to CCHFV. Despite being classified as a biosafety level (BSL) 3 pathogen, DUGV causes only mild to no clinical symptoms in both animals and humans. Therefore, it is seen as highly understudied due to its perceived low relevance. Confirmed reports of virus detection in ixodid ticks (mostly collected from cattle) has been limited to West and Central African countries as well as Uganda [10]. On the other hand, NSDV is known to be a devastating agent affecting small ruminants, causing hemorrhagic gastritis and abortions in sheep and goats with a lethality rate of up to 90% thus being considered a BSL 3 pathogen. Similar to DUGV, human infection with the virus can lead to a (mild) febrile illness including nausea and headache. Until now, the virus has been described in India, Sri Lanka, China and East Africa [11]. Data on both viruses in Mozambique are rather patchy or non-existent, respectively.

The main objective of this study was to find the first proof of CCHFV in animals or ticks, which should contribute to filling the gaps of knowledge of the virus distribution in Mozambique/southern Africa. For this purpose, serum samples and ticks were collected from cattle in the area of the Limpopo National Park (LNP) in the Gaza province at the South African border and subsequently analyzed using serological (ELISA) and molecular methods (PCR). Positive samples were intended to be sequenced and further characterized in order to identify which CCHFV strain(s) are circulating in the country. Since limited information is known about the distribution of DUGV and NSDV, the collected ticks were also to be examined for these viruses in order to obtain possible evidence of the pathogens outside the known distribution areas. In addition, the serum samples collected were also tested for RVFV antibodies, as the last RVFV outbreak in the Mozambique in 2014 [7] occurred close to the collection sites of this study.

## Materials and methods

### Sampling region

The LNP is still relatively young being founded in 2002 and forms along with the Gonarezhou National Park in Zimbabwe (GNP) as well as the well-known Kruger National Park (KNP) in South Africa the Great Limpopo Transfrontier Park. The park itself is located in the southwest of the country in the Gaza province. Due to poaching and the turmoil of the Mozambican Civil War (1977–1992), almost all wildlife living in the present LNP was eliminated. Only in recent years, up to 5000 wild animals were translocated from the KNP to the LNP and the border fence in the area was partially dismantled [12] allowing wild life to cross between the parks. Despite the status being a nature reserve, there is a considerable number of people living with their livestock (cattle, sheep and goats) in the buffer zones, but also in central sections of the LNP [13]. Combined with the border location of the LNP, these factors make the region a very interesting study area in terms of interactions between humans, livestock and wild life. The vegetation of the Limpopo National Park and the surrounding buffer zone is characterized by a mixture of savannah, wetlands, forests and riverine woodlands. Sampling was carried out during the dry season, which lasts from April to November, when the roads are usually in better condition and communities more accessible. During this season, the rivers and pastures were dry and the animals were forced to seek out distant grazing areas and water sources. Soil conditions vary throughout the park, with sandy, well-drained soils in some areas and more fertile soils near the banks of the Elephant River favoring dense vegetation.

### Animal samples collection

**Fig. 1:**
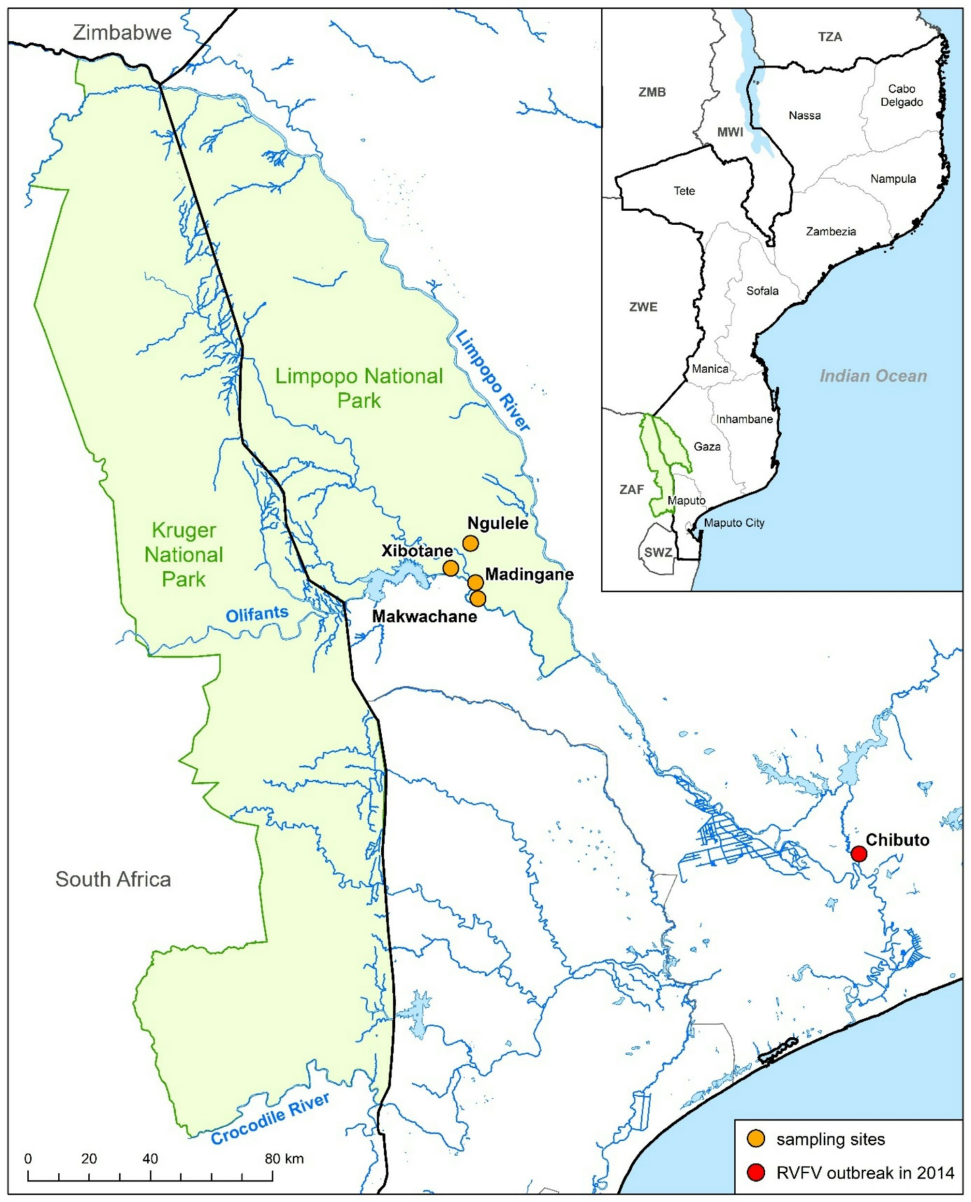
Map of Mozambique and the border region of South Africa showing the Limpopo National Park and the Kruger National Park situated along the border (Nguele (-23.78231; 32.28868), Xibotane (-23.8826; 32268), Madingane (-23.87769; 32.30089), and Makwachane (-23.94382; 32.27951)). The map was created using ArcGIS ArcMap 10.8.1 (ESRI, Redlands, CA, USA) along with GIS data from GADM data (2018–2022 GADM; https://gadm.org/license.html) and OpenStreetMaps (OpenStreetMap contributors; https://www.geofabrik.de/de/data/download.html; https://wiki.openstreetmap.org/wiki/DE:Legal_FAQ)

A multistage sampling approach was employed. Villages were first selected based on geographic distribution, and within each village, households were randomly selected. Overall, samples were collected at four different villages/communities (Fig. 1): Nguele (-23.78231; 32.28868), Xibotane (-23.8826; 32268), Madingane (-23.87769; 32.30089), and Makwachane (-23.94382; 32.27951). Within the selected households, animals were examined for the presence of ticks, and only those with ticks were sampled. The approach focused on tick-infested animals to maximize the likelihood of detecting tick-borne viruses. As a result, 460 cattle blood samples and 1176 ticks were collected between 2019 and 2020 directly from tick-infested animals. Prior to sampling, the objectives and procedures of the study were explained to the farmers. The local leaders gave their verbal consent and invited the farmers (randomly selected) to participate in the study. Samples were randomly collected from the cattle pens based on the consent of the owners, the condition of the pens (which were often of simple/improvised construction material) and the presence of ticks. Samples were taken from animals of all ages and sexes, most of them from the ‘Landim breed’ (a local breed). Blood samples were collected from the coccygeal vein in plain serum-separating tubes. Clotted blood samples were obtained by centrifugation and the sera was stored at -20^o^C until use. The collection of ticks in cattle was performed by examining specific body sites known to harbor these arthropods (ear, dewlap, shoulder, belly, groin, udder and perineum). No field variables such as ‘climate’ or ‘weather’ were recorded.

### Serology

Serum samples were tested at the Eduardo Mondlane University for CCHFV and RVFV antibodies using ID Screen CCHF Double Antigen Multi-species (IgM/IgG) and ID Screen Rift Valley Fever Competition Multi-species commercial ELISA test kits (IDvet, Grabels, France), respectively, according to manufacturer’s instructions. Both tests target the anti-nucleoprotein (NP) antibodies of CCHFV and RVFV. Optical readings were made at 450 nm, the results expressed as percentage of high positive control and the cut-offs suggested by the manufacturer were followed.

### Tick screening

Ticks were morphologically identified under a stereo microscope using the identification keys of Apanaskevich et al. [14, 15]. All ticks were individually homogenized in AVL-buffer followed by an automatic nucleic acid extraction using a KingFisher Flex (ThermoFisher, Waltham, USA) device [16]. Subsequently, the samples were analysed individually with different RT- qPCRs assays for CCHFV [17], NSDV [11] and DUGV [10]. Additionally, selected samples were screened with a pan-Flavivirus melting curve PCR [18].

### Statistics

Seroprevalence for CCHFV and RVFV was determined for each sampling site, age group, and sex, using the proportion of positive cases relative to the total number of samples in each subgroup. Seroprevalence estimates were calculated with 95% confidence intervals (CIs).

Univariable logistic regression was conducted for each factor (age, sex, and site) to assess their association with seroprevalence for both CCHFV and RVFV. Odds ratios (ORs), 95% CIs, and p-values were calculated for each factor.

A generalized mixed-effects linear model (GLMM) was used to examine the effects of age, sex, and site, as well as interactions between these variables. Interaction terms for age × site and sex × site were included to assess whether the associations varied by site. The model was structured as follows:

$$\begin{array}{l}\:{\rm{log}}\left( {\frac{\pi }{{1 - \pi }}} \right) = {\beta _0} + {\beta _1} \cdot \:age + \:{\beta _2}.\:sex\\\,\,\,\,\,\,\,\,\,\,\,\,\,\,\,\,\,\,\,\,\,\,\,\,\, + \:{\beta _3}.site\: + \:{\beta _4}.\left( {Age \times \:site} \right) + {\beta _5}.\left( {Sex \times \:site} \right)\:\:\end{array}$$

Statistical analysis was conducted with the SPSS software version 22.0 for Windows (IBM Corp., New York, NY, USA). A p-value < 0.05 was considered statistically significant.

## Results

### Serology

Four hundred and sixty cattle serum samples from four different sampling sites were screened for CCHFV and RVFV antibodies revealing a seroprevalence of 50% and 28%, respectively (Table 1). The results from the GLMM analysis indicated that the model was statistically significant for both CCHFV and RVFV (*p*-value < 0.05). Table 2 presents the *p*-values and Odds Ratios for each of the risk factors analyzed in this study. More detailed information and all model outputs are shown in Suppl. Tables 1 and 2. For CCHFV, age and sex were identified as significant risk factors, with *p*-values of 0.001 and 0.013, respectively. In contrast, sampling sites did not show statistical significance (*p* > 0.073). The odds ratio for sex (OR = 0.641) suggests a lower likelihood of CCHFV positivity in females compared to males. Significant seroprevalence differences between age groups were only observed for CCHFV in three out of four sampling sites. Hereby, more animals tested seropositive were found in the older age groups compared to the younger ones (Tables 1 and 3). For RVFV, neither age (*p* > 0.650) nor sex (*p* = 0.914) were significant predictors, but sampling sites demonstrated a statistically significant effect (*p* = 0.001). In Makwachane and Xibotane, significantly more animals were seropositive than in the other two collection sites (Tables 1 and 2).

### Tick screening

In total, 1176 ticks were examined. The vast majority of them belonged to the genus *Hyalomma* (*Hyalomma (H.) rufipes*, *n* = 1071; *H. truncatum*, *n* = 78), followed by a few *Amblyomma (A.) hebraeum*, *n* = 21) and *Rhipicephalus* species (*n* = 6) ticks. All ticks were negative for CCHFV, DUGV and NDSV in RT-qPCR. In addition, 588 ticks were tested for Flaviruses. PCR amplicons of three potentially positive samples samples were sequenced via Sanger sequencing (Eurofins) but no specific Blast results were obtained.

**Table 1:** CCHFV / RVFV seroprevalences and differences between the age groups in cattle of all four surveyed sampling sites in Limpopo National park (95% confidence interval (CI %) in brackets)

Sampling sites	CCHFV			RVFV		
	*n*	*p*	Prev. (%)	*n*	*p*	Prev. (%)
Makwachane	110	43	39.1 (45.3–54.7)	110	57	51.8 (42.1–61.4)
Xibotane	88	46	52.3 (41.4–63.0)	89	51	57.3 (46.4–67.7)
Madingane	224	120	53.6 (46.8–63.0)	182	15	8.2 (4.7–13.2)
Ngulele	38	21	55.3 (38.3–71.4)	79	8	10.1 (4.5–19.0)
Total	460	230	50.0 (45.3–54.7)	460	131	28.5 (24.4–32.8)
**Age group**	**n**	**p**	**Prev. (%)**	**n**	**p**	**Prev. (%)**
0-2y	56	9	16.7 (7.6–28.3)	61	21	34.4 (22.7–47.7)
3-4y	128	35	27.3 (19.8–35.9)	132	42	31.8 (24.0- 40.5)
5-6y	198	127	64.1 (57.0-70.8)	192	46	23.9 (18.1–30.6)
> 6y	78	59	75.6 (64.6–84.7)	75	22	29.3 (19.4–41.0)

n = number of tested animals

p = number of positive animals

Prev.= prevalence

**Table 2:** Risk factor analysis for CCHFV and RVFV (Odds ratios (ORs), confidence intervals (CIs), and p-values)

Risk Factors	Level	Reference Level	CCHFV OR (95% CI)	CCHFV *p*-value	RVFV OR (95% CI)	RVFV *p*-value
**Age**	0–2 years	> 6 years	0.20 (0.09–0.43)	< 0.001	1.18 (0.59–2.33)	0.897
	3–4 years	> 6 years	0.32 (0.18–0.57)	< 0.001	1.05 (0.57–1.96)	0.867
	5–6 years	> 6 years	0.85 (0.53–1.36)	0.480	0.75 (0.38–1.46)	0.650
**Sex**	Male	Female	0.68 (0.48–0.97)	0.013	1.02 (0.67–1.56)	0.914
**Sampling site**	Makwachane	Ngulele	0.89 (0.53–1.49)	0.480	5.12 (2.12–12.37)	< 0.001
	Xibotane	Ngulele	1.56 (0.95–2.56)	0.073	6.03 (2.52–14.45)	< 0.001
	Madingane	Ngulele	1.20 (0.70–2.08)	0.235	0.75 (0.28–1.98)	0.573

**Table 3:** Comparison of the different age groups in terms of CCHFV Seroprevalence from the three regions where a significant pattern of age distribution was observed (significant p-value < 0.05)

Region	Differences between age groups		*P*-value
**Madingane**	0–2 0–2 0–2 3–4 3–4 5–6	3–4 5–6 > 6 5–6 > 6 > 6	0.967 < 0.001 < 0.001 < 0.001 < 0.001 0.434
**Makwachane**	0–2 0–2 0–2 3–4 3–4 5–6	3–4 5–6 > 6 5–6 > 6 > 6	0.938 < 0.001 < 0.001 < 0.01 < 0.001 0.639
**Xibotane**	0–2 0–2 0–2 3–4 3–4 5–6	3–4 5–6 > 6 5–6 > 6 > 6	0.961 < 0.05 0.142 < 0.001 0.096 1.000

## Discussion

Mozambique in southeastern Africa is considered under-studied in terms of the knowledge of CCHFV distribution and occurrence. The same applies to large parts of the African continent. However, it must be assumed that the virus is endemic in many parts of the continent, but has remained unrecognized due to a lack of awareness and testing capacities. It is only in the last year that the first confirmed records of the virus have been reported in Mozambique’s northern neighbors Malawi [19] and Zambia [20]. IgG antibodies in human patients from Mozambique were first detected in 2015. By examining the bovine sera and ticks that were collected, we aimed to prove the (very likely) presence of CCHFV in host animals and/or reservoirs in Mozambique. Thus, it was not surprising that CCHFV IgG antibodies were found in 50% of the cattle tested. To our knowledge, this is the first detection of CCHFV antibodies in animals in the country. These high seroprevalences were generally in line with recent data [19, 21] from cattle in South Africa (76.7%) and Malawi (46.9%). As described previously [2, 22–25], we also observed a significant effect of age on CCHFV seroprevalence in three out of four surveyed sampling sites (Tables 2 and 3). Compared to younger, naive individuals, older animals are more likely to have come into contact with CCHFV-positive ticks in the course of their lives and therefore often show a higher seroprevalence. The lack of age impact in the Ngulele sampling site might be a consequence of the small number of sampled animals in the respective age groups (e.g. in group " >6” were only five animals). Although adequate numbers of male (*n* = 156) and female (*n* = 304) cattle were sampled overall, no impact of sex on the CCHFV prevalence status could be observed. Assuming that male and female animals are equally herded, they share the same risk of exposure to ticks, hence the same risk of contracting the virus. Since cattle-wildlife contacts are likely to occur in the LNP, it would also be interesting and advisable to sample game to learn more about the mutual interactions between livestock and wildlife. A study from Kenya published in 2021 revealed an extremely high CCHFV seroprevalence (92.1%) in African buffalo (*Syncerus caffer*) in populations without contact with livestock, whereas the seroprevalence was significantly lower while sharing their habitat with domestic cattle [26]. However, it is currently unknown which factors play a major role in this context, as only limited data on wildlife-livestock interactions and the associated infection dynamics are available to date. Regardless of this high seroprevalence in cattle, the fact that only one human case has been described in the country so far [3] is not a contradiction per se, but reflects exactly what is already known about CCHFV. Given the very wide distribution of the virus (at least in rural endemic areas), there are comparatively few clinical cases in humans - presumably because most infections are subclinical and do not require further treatment, and because only certain professions/ groups of people are usually affected (farmers, slaughterhouse workers, healthcare workers, etc.). It is also important to emphasize that the virus often occurs in regions where testing capacities are limited, which can lead to misleading conclusions in the interpretation of clinical data [1].

Despite 50% seropositive animals, the virus itself could not be found in any of the examined ticks. The ticks were collected mainly in April and December, which means that a potential seasonal pattern of the occurrence of tick species and their infection status would not have been captured. The ticks were also negative for DUGV, NSDV and Flaviviruses. In this regard, besides seasonality, the tick species might have also played an important role. The vast majority of screened ticks belonged to the genus of *Hyalomma*. So far, transovarial and transstadial transmission of DUGV has only been demonstrated in *Amblyomma variegatum* [27] while the virus was also found in other tick species/genera [28]. For NSDV, *Rhipicephalus appendiculatus* in Africa and *Haemaphysalis intermedia* in Asia are mainly considered as the main vector and reservoir [29]. Therefore, future tick surveillance studies should be conducted more broadly throughout the year and other tick species should be also targeted for collection, especially if DUGV and NSDV are considered for investigation. Since the samples originated from ticks collected from the host, future studies should also include unfed ticks collected directly from the environment, which can provide a more accurate image of the actual prevalence in the ticks.

Previous RVFV studies in cattle in Mozambique revealed overall seroprevalences of 37.3% [8] and 36.9% [9], respectively. These findings were slightly higher compared to the prevalence reported in the present study of 28.5%. However, the results in 2017 [9] from the province of Gaza (Chóckwè, Mabalane and Bilene districts) revealed a seroprevalence of 19.2%, which is slightly lower than the results of this current study. Unfortunately, it is not clear from this manuscript how high the individual seroprevalences were. In this context, the results from Mabalane, which is fairly close to the collection sites inside the LNP, would have been interesting to compare. On the other hand, there was a notable difference (Table 1) between the sampling sites Makwachane (51.8%) and Xibotane (57.3%) versus Madingane (8.2%) and Ngulele (10.1%). The village of Ngulele is a small community that is very far away from other localities. They operate a commercially oriented farm with more favorable husbandry conditions. In contrast to other communities, they have the necessary resources to obtain medication and have access to a veterinarian. The cattle in Madingane do not share the same grazing area with the other herds, as they are located in a low-lying area. This natural geographical barrier means that they cannot interact with animals from other places. However, when looking at satellite images of the sampling sites, it is noticeable that “Makwachane” and “Xibotane” are only a few hundred meters away from the Elefantes River, while “Madingane” and “Ngulele” are over a kilometer away from the nearest larger river. Such a geographical situation could be a major factor favoring the breeding of mosquitoes, the vector of RVFV. Unfortunately, there is no information about the origin of the animals (trade, purchases etc.) and how all four sampling sites differ in terms of the exact ecology (presence of still/stagnant water etc.), which have a significant impact on mosquito propagation and thus also on RVFV maintenance [5]. While there was a significant age dependency in CCHFV, no age effect was found at all for RVFV (Tables 1 and 2). Unlike CCHFV, which poses a nearly all-season threat due to latently infected ticks, RVFV outbreaks occur more sporadically. These outbreaks are usually very severe and a large number of animals of all ages can become infected in a short period of time. However, there has been no large RVF outbreak in the Gaza province (Fig. 1) in recent years since 2014 [7]. The fact that we have found a relatively high seroprevalence may have several reasons. First of all, this suggests that there is probably an endemic/silent RVFV cycle in the region that may be responsible for the seroprevalence found in cattle. Secondly, (adult) cattle are less likely to show symptoms than small ruminants, which means that sporadic infections can occur that are not recognized. In addition, as cattle are much older on average, herds at an older age may be better protected by antibodies than herds of small ruminants. As a result, potential outbreaks or sporadic diseases may be more difficult to detect. Since no RVF vaccination has been carried out in the region of the sampling sites, this points to a constant (low- level) and silent RVFV circulation, which was also already assumed in previous literature on RVFV in Mozambique [5]. The uniformly high RVFV seroprevalence in all age groups is also indicative of a recent, undetected outbreak event. Although no human cases of RVFV have been reported recently, farmers and slaughterhouse workers in the region should be sensitized when handling animal products (carcasses, blood, amniotic fluid/ abortions, etc.) as this represents the main transmission route for humans.

The multivariable model in this study included only sex, age group, and location due to the limited availability of data on animal exposure and livestock keeping practices. We recognized that these factors could potentially influence seropositivity rates for CCHFV and RVFV. Future studies should aim to collect more detailed data on livestock ownership, husbandry system, animal contact, and environmental exposures etc. in order to improve the model’s ability to detect associations with seropositivity [30]. The small sample sizes in some of the villages may have contributed to the lack of statistical power needed to detect consistent associations across sampling sites.

## Conclusion

This study revealed the presence of anti-CCHFV and anti-RVFV specific antibodies in cattle sampled in four locations in the LNP suggesting that those viruses are actively circulating in the park and may be an important but overlooked cause of non-identified febrile illness in humans. As no RVFV outbreaks have been reported in the region since 2014, the high seroprevalence suggests that this involves a silent RVFV cycle and/or that undetected outbreaks may have occurred. Especially for naive herds and the more susceptible small ruminant species, there is an increased risk of an acute outbreak with potential animal losses. Therefore, active surveillance of livestock in this area should be increased and livestock farmers sensitized to this risk. Since there is still no direct evidence of CCHFV in the country, the molecular detection of the virus in ticks and/or their hosts, including genetic characterization, would be an important piece of the puzzle for a better understanding of its circulation in Mozambique.

## Electronic supplementary material

Below is the link to the electronic supplementary material.

Supplementary Material 1

## Data Availability

No datasets were generated or analysed during the current study.
