# Supplementary material for: Zoonotic arbovirus infections in cattle in Mozambique with special reference to Crimean-Congo hemorrhagic fever virus (CCHFV) and Rift Valley fever virus (RVFV)
Source: Virol J. 2025 Jun 6;22:185. doi: 10.1186/s12985-025-02804-9 (PMC12144736; doi:10.1186/s12985-025-02804-9)
Supplement: Supplementary file 1 — Supplementary Material 1 [file 12985_2025_2804_MOESM1_ESM.pdf]

UNIVERSIDADE EDUARDO MONDLANE

Centro de Biotecnologia

CB · UEM

Dear Farmer

The Biotechnology Center and the Veterinary Faculty of the Eduardo Mondlane University, together with the Friedrich-Loeffler-Institut (Federal Research Institute for Animal Health, Germany), are conducting a study on cattle, goats and sheep to determine whether Rift Valley Fever and Crimean-Congo Hemorrhagic Fever occur in these animals. These diseases are not only important in animals but also in humans, and can cause symptoms similar to malaria and, in severe cases, can cause death.

As there is not much information regarding these diseases in the areas located in the Limpopo National Park and surrounding areas, the three institutions decided to study the presence of these diseases in this location.

The study was duly authorized by the Scientific Committee of the Mozambican institutions and the collection of samples from the animals will be carried out by specialized personnel, to avoid discomfort/injury to the animals, in compliance with rules established in the country and internationally. The study will also be monitored by the local veterinary authorities (District Services for Economic Activities) who have assigned a technician to work with our team. The technicians will collect approximately 5 ml of blood from each animal and tick and will ask questions to determine whether there are clinical signs related to the diseases under study. The results of this study will help to identify the existence of this disease in the localities and to establish preventive measures to avoid future cases of disease in animals and humans.

We therefore request your authorization to collect samples from your animals and would like to thank you in advance for all your collaboration.

Maputo, April 2025

Dr. Lucinda de Araújo

Av. de Moçambique, Km 1,5, C. Postal 257, Tel.: (+258) 21 477227, Fax.: (+258) 21 477227, www.cb.uem.mz
Maputo – Moçambique
